# Binge alcohol and HIV: leaky gut and neurodegeneration through the gut–brain axis

**Published:** 2019-04-30

**Authors:** Musthafa Mohamed Essa, Byoung-Joon Song, Sulie L. Chang

**Affiliations:** 1 Department of Food Science and Nutrition, College of Agricultural and Marine Sciences, Sultan Qaboos University, Muscat, Oman; 2 NIAA, NIH, Rockville, MD; 3 Institute of Neuroimmune Pharmacology, Seton Hall University, South Orange, NJ, USA; 4 Department of Biological Sciences, Seton Hall University, South Orange, NJ, USA

Currently, approximately 1.2 million people in the United States and 36.7 million individuals globally^[1,2]^ are living with human immunodeficiency virus (HIV) infection. Unfortunately, around 20% of HIV-infected people do not know whether they are infected with HIV^[1]^ and continue unhealthy lifestyles of tobacco smoking, binge alcohol drinking, and intake of n-6 fatty acid-rich junk foods. In addition, binge drinking is now considered as a major public health problem and accounts for more than 75% of alcohol consumed in the United States.^[3]^ Furthermore, alcohol abuse is a major cause of various disease states frequently observed in HIV-infected people^[4]^ as approximately 50% of these HIV-infected people often indulge in binge drinking.^[5]^

Binge alcohol consumption is also considered one of the risk factors for HIV infection^[1]^ and excessive alcohol intake could accelerate the progression of acquired immunodeficiency syndrome (AIDS).^[6]^ Binge drinking could increase the risk of tissue injury in many organs. In the liver, it can cause severe hepatitis, fibrosis, cirrhosis, and carcinogenesis with liver failure, which is considered one of the main causes of death in HIV-infected people.^[3–5]^ In the brain, excessive alcohol intake can cause Wernicke-Korsakoff syndrome, fetal alcohol syndrome, cerebellar degeneration, and dementia. Elevated bacterial endotoxin, including lipopolysaccharide from gut leakage, has been reported to play a critical role in the development and progression of both alcoholic^[7]^ and nonalcoholic^[8]^ inflammatory tissue injuries. Recent reports suggest that HIV-infected people with suppressed replication of HIV-1 due to treatment with the highly active antiretroviral therapy^[9,10]^ are more sensitive to alcohol-induced gut leakiness, resulting in inflammatory tissue injury. It is important to emphasize that HIV infection is also thought to change intestinal microbial composition and increase gut leakiness, prior to manifestations of AIDS-associated chronic inflammation and more severe symptoms, such as HIV-associated cognitive disorders (HAND) and HIV-associated dementia (HAD).^[11,12]^ However, the molecular mechanisms for intestinal microbiome change, elevated gut leakage, and advanced inflammatory disease observed in HIV-infected people (and rodents) are not well understood.

Elevated neuronal death and impaired cognition are common outcomes of alcohol abuse and HIV-1 infection.^[13]^ Studies suggest that the onset and progression of HIV-associated neurological disorders could be highly impacted by binge drinking.^[14]^ In addition, abused drugs (like morphine) are highly common during excessive drinking, which further leads to the accelerated progression of opioid use disorders and tissue injury.^[15]^ Combined effect of HIV and binge drinking result in excessive production of free radicals, including reactive oxygen and nitrogen species, which can enhance mitochondrial dysfunction, energy imbalance, and necroapoptosis of parenchymal cells, eventually contributing to leaky gut, endotoxemia, neuroinflammation, tauopathy, and neurotoxicity. Increased neuroinflammation and neuroimmune signaling are highly linked with neuronal dysfunction.^[16,17]^ The level of interleukin (IL)-18, a proinflammatory cytokine produced in microglia of brain, was elevated in HIV-1-infected people.^[18,19]^ In addition, increased IL-18 and other proinflammatory cytokines/chemokines are highly associated with neuroinflammation followed by motor and cognitive dysfunction.^[14]^ All these alterations likely lead to activation of glial cells and astrocytes followed by increased neuronal cell death with impaired cognition and behavior, ultimately contributing to neurodegeneration such as HAND and HAD [Figure 1]. The mechanisms by which alcohol, abused drugs, and HIV-1 infection promote gut leakage and further neurodegeneration are not well defined. We believe that this area needs to be extensively studied to elucidate the gut–brain interactions and generate the lead compounds for novel therapeutic strategies against pathologies associated with HIV and binge alcohol drinking alone or in combination.

Numerous reports conducted in both animals and humans demonstrated that alcohol consumption, one of the widely abused substances, elevates the AIDS-related disease progression. For instance, preclinical and clinical studies showed a direct correlation between alcohol abuse and escalation of the HIV-associated neurodegeneration. Binge alcohol intake is likely to directly promote neuroinflammation and neurodegeneration in HIV-infected individuals as well as indirectly through the leaky gut and endotoxemia. Oxidative/nitrosative stress and the possible involvement of tryptophan metabolites associated with neuroinflammation might be major contributors to HAND and HAD in HIV-infected people especially with binge alcohol consumption. Based on these mechanistic studies on the HIV-associated disease states, numerous investigators have recently started studying the benefits of natural molecules, including n-3 fatty acids, against these ailments. Supplementation with docosahexaenoic acid and eicosapentaenoic acid could be able to reduce the amounts of IL-6, IL-18, and nuclear factor-B in the striatum and pave a novel way to alleviate the additive or synergistic effects of HIV infection and binge drinking.^[14]^ Dietary supplementation with n-3 fatty acids and/or some polyphenols, including ellagic acid, urolithin, curcumin, or quercetin, may have beneficial effects on the development and progression of HAND/HAD. To find out the exact mechanism(s) of action, extensive preclinical and clinical studies are warranted.

## Figures and Tables

**Figure 1:**
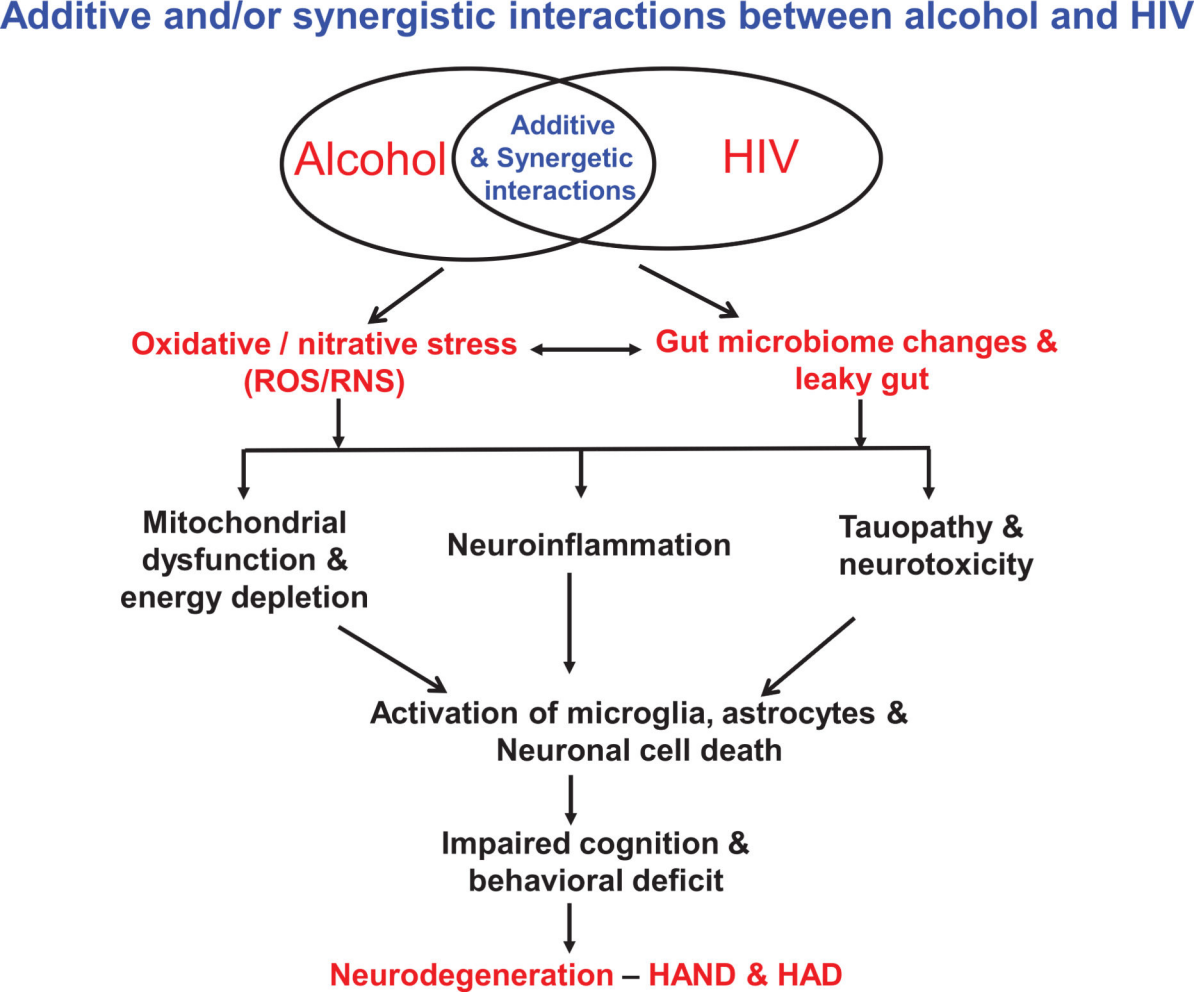
Schematic diagram of neurodegeneration through the potential additive and synergetic interactions between alcohol and HIV infection.
